# Scale-up of reverse electrodialysis for energy generation from high concentration salinity gradients

**DOI:** 10.1016/j.memsci.2021.119245

**Published:** 2021-06-01

**Authors:** A.M. Hulme, C.J. Davey, S. Tyrrel, M. Pidou, E.J. McAdam

**Affiliations:** Cranfield Water Science Institute, Cranfield University, Bedfordshire, MK43 0AL, UK

**Keywords:** Closed-loop, Recycle, Battery, Thermal-to-electric, Cell pair, Salinity gradient energy

## Abstract

Whilst reverse electrodialysis (RED) has been extensively characterised for saline gradient energy from seawater/river water (0.5 M/0.02 M), less is known about RED stack design for high concentration salinity gradients (4 M/0.02 M), important to closed loop applications (e.g. thermal-to-electrical, energy storage). This study therefore focuses on the scale-up of RED stacks for high concentration salinity gradients. Higher velocities were required to attain a maximum Open Circuit Voltage (OCV) for 4 M/0.02 M, which gives a measure of the electrochemical potential of the cell. The experimental OCV was also much below the theoretical OCV, due to the greater boundary layer resistance observed, which is distinct from 0.5 M/0.02 M. However, negative net power density (net produced electrical power divided by total membrane area) was demonstrated with 0.5 M/0.02 M for larger stacks using shorter residence times (three stack sizes tested: 10 × 10cm, 10 × 20cm and 10 × 40cm). In contrast, the highest net power density was observed at the shortest residence time for the 4 M/0.02 M concentration gradient, as the increased ionic flux compensated for the pressure drop. Whilst comparable net power densities were determined for the 10 × 10cm and 10 × 40cm stacks using the 4 M/0.02 M concentration gradient, the osmotic and ionic transport mechanisms are distinct. Increasing cell pair number improved maximum current density. This subsequently increased power density, due to the reduction in boundary layer resistance, and may therefore be used to improve thermodynamic efficiency and power density from RED for high concentrations. Although comparable power densities may be achieved for small and large stacks, large stacks maybe preferred for high concentration salinity gradients due to the comparative benefit in thermodynamic efficiency in single pass. The greater current achieved by large stacks may also be complemented by an increase in cell pair number and current density optimisation to increase power density and reduce exergy losses.

## Highlights

• RED for energy generation from high concentration salinity gradients in studied.
• Boundary layer effects require higher velocities to achieve maximum OCV.
• Highest power at short residence time with larger stack contrasting sea/riverwater.
• Same power density for small and large stacks, but higher current in large stack.
• More cell pairs lowers boundary layer resistance and improves power density.

## Graphical abstract

**Figure:**
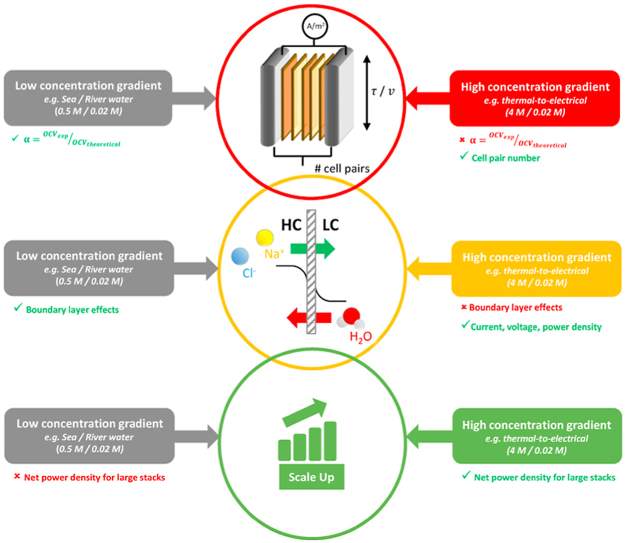

## 1 Introduction

Electricity consumption has increased to unprecedented levels due to worldwide population and economic growth [1], which has accelerated national decarbonisation strategies to mitigate the effects of global warming [2]. This requires innovative solutions to produce ‘green’ energy in addition to technologies that can store energy from transient sources such as wind and solar in order to operate synergistically to sustain the base load supply from renewables. Waste heat can be considered one relatively underexploited ‘green’ opportunity to meet electrical energy demand through thermal-to-electrical conversion, since around 246 PJ waste heat is available from industrial, residential and transportation sectors [3]. However, the majority of this heat energy is classified as low-grade heat (<100 °C) [3] which is not conducive to the use of organic rankine cycle [4] or thermoelectric generators [5] due to their high cost and low efficiencies within this domain. The reverse electrodialysis heat engine (RED-HE) has been theoretically demonstrated to obtain up to 85% exergy efficiency using equivalent heat sources [6]. Low-grade waste heat is used in a distillation process to generate two solutions of different salinities [7,8]. The Gibbs free energy of mixing these two solutions across a reverse electrodialysis (RED) stack can then produce power by facilitating ionic transport across alternately arranged anion and cation exchange membranes which is subsequently converted to an electrical current by a redox couple circulating across the electrode [9]. An analogous closed-loop RED configuration has been similarly demonstrated for energy storage [10], which implies that the same technology could respond to multiple demands underpinning the decarbonisation agenda.

Whilst RED has been demonstrated to produce high exergy efficiency at laboratory scale [11], successful implementation of RED technology requires that the power production estimated at laboratory scale can be realised following translation to full-scale, which includes matching the current and voltage specified. A single stack can be scaled up by increasing cell pair number [12] or increasing stack membrane dimensions (L x W), where both strategies increase total membrane area to deliver higher total power [9]. Veerman et al. [13] provided the first study to demonstrate power production from a demonstration scale RED (stack size 25 × 75 cm), with a total membrane area of 18.75 m^2^ (Table 1). While data from several further demonstration scale RED stacks have since been published (Table 1), the impact of scaling-up stack size via cell pair number or membrane area on power density and energy efficiency has not been extensively characterised. The greater membrane area associated with an increase in cell pair number provides an improvement in power output, with an increase in cell pair number from 5 to 50 pairs in a typical laboratory stack size (10 × 10 cm) demonstrated to provide a proportionate increase in total power using artificial feeds with equivalent concentrations to sea and river water [12]. Comparable power density has also been observed for a 10 × 10 cm laboratory stack despite differences in cell pair number and membrane type, by fixing residence time across the different stack compositions. Moreno et al. [14] was similarly able to demonstrate comparable power density and energy efficiency across four stack sizes ranging from 6 × 6 cm to 44 × 44 cm by fixing residence time as a constant. In addition, to residence time, fluid velocity must also be considered in stack design, in order to minimise concentration polarisation. In a square stack, flowrate must be increased four-fold to match residence time when membrane length scale is doubled, which subsequently doubles velocity. The comparable power density observed would indicate similar resistance between stack sizes, where the increase in velocity compensates for the further axial development of the boundary layer imposed by extended membrane length. However, at constant velocity, the authors noted an increase in gross energy efficiency with larger stack sizes. To fix velocity, a two-fold increase in flow rate must be applied when the length scale of a square stack is doubled, which subsequently doubles residence time. It was therefore proposed that the improved exergy conversion was due to the increased ionic transport experienced when extending fluid residence time.

**Table 1:** Summary of all scaled-up RED systems in the literature.

Feeds	Stack dimensions (cm x cm)	Cell Pair No.	Total membrane area (m^2^)	Spacer thickness (μm)	Feed Temp. (^o^C)	Feed Velocity (cm s^−1^)	Power Density (W m^−2^)	Power (W)	Ref
HC 0.51 M NaCl LC 0.017 M NaCl	25 × 74	50	18.75	200	25	0.1	0.62	–	[13]
HC 0.51 M NaCl LC 0.017 M NaCl	10 × 10	10–50	0.2–1	200	25	1	0.93	0.2–0.93	[12]
HC 0.48 M NaCl LC 0.003–0.009 M NaCl					–	–	–	Aim: 50 kW	[15]
HC 0.5 M NaCl HC 5 M NaCl	20 × 20	100	8	270	20	3.5	0.85	–	[16]
LC 0.1 M NaCl HC 5 M NaCl					40	3	3.0	–	
LC 0.03 M NaCl^a^ HC 4–5 M NaCl^a^	44 × 44	125	48	280	17–31	1	0.8	40	[17]
LC 0.03 M NaCl^b^ HC 4–5 M NaCl^b^					25–28	1	1.4	65	
LC 0.007–0.06 M NaClc HC 4 M NaClc	44 × 44	1 × 125d 2 × 500d	>400	280	17–31	0.5–0.9	1.7	330	[18]
LC 0.007–0.06 M NaClb HC 4 M NaClb					25	0.9	2.1	700	
LC 0.01–0.06 M NaCl^e^ HC 0.5 M NaCl^e^	43 × 29	1000	250	100	16–22	1.5	0.38	95	[19]
LC 0.017 M NaCl HC 0.51 M NaCl	6 × 6 10 × 10 22 × 22 44 × 44	50	0.36 1.00 4.84 19.36	0.155	25	0.25–2	1.4		[14]

– Indicates where no data is available. ^a^Concentrated brine and brackish water with a conductivity equivalent to these concentrations. ^b^Artificial solutions with a conductivity equivalent to these concentrations. ^c^Concentrated brine and brackish water. ^d^Modules operated in parallel. ^e^Municipal wastewater and seawater with a conductivity equivalent to these concentrations.

Whilst evidence for the scalability of RED is encouraging, these studies all employed sodium chloride feeds with a fixed concentration equivalent to seawater/river water, where the electrochemical potential resulted in a maximum reported power density of 0.93 W m^−2^ [12]. Artificial saline solutions can instead be used in closed-loop applications, such as energy storage and thermal-to-electric conversion, to increase the concentration gradient and improve power densities up to 6.7 W m^−2^ for the same salt [20]. The use of ‘ideal salt solutions’ is made possible through regeneration within the closed-loop, resulting in negligible fouling, and the presence of only monovalent ions, which presents markedly different implications for scale-up relative to sea water/river water where extensive pre-treatment is required to prevent fouling. However, the elevated concentration gradient establishes a greater osmotic gradient. This promotes unfavourable osmotic water transport [21], which we propose can increase upon scale-up since water transport is generally scalable to membrane area. Concentration polarisation may also be exacerbated for larger membrane areas, due to the axial development of a more concentrated boundary layer induced by the high concentration gradient [22]. To illustrate, Tedesco et al. [17] obtained power densities up to 4 W m^−2^ using 5 M and 0.5 M sodium chloride (NaCl) feeds at 40 °C in a 10 × 10 cm stack. However, increasing the stack size to 20 × 20 cm and doubling the cell pair number at constant velocity reduced power density. Energy efficiency was also similar at both process scales, despite an eight-fold increase in residence time. This suggests that the approach to scale-up of RED for high concentration salinity gradients may be distinct and contradicts observations made for low concentration salinity gradients (seawater) [14].

In RED, energy efficiency is defined as the percentage of available Gibbs free energy that is transformed into power production [9]. For closed-loop RED, the systems level efficiency is also determined by solution regeneration efficiency. For example, thermal utilisation efficiency [7] in the RED heat engine relates the thermal energy required for solution regeneration to the electrical energy generated by RED. This implies that energy extraction from the finite volume of regenerated working solution must be maximised to reduce unused exergy leaving the stack and improve the thermodynamic efficiency [14]. This can be facilitated by increasing cell pair number [12], increasing residence time [14] or introducing feed solution recycle which has been demonstrated to improve RED energy efficiency at lab-scale [11]. However, for high concentration salinity gradients, the cumulative effect of recycle may increase concentration polarisation and osmotic water transport phenomena, which must be managed to sustain power output and energy efficiency. Since these phenomena occur concomitantly, the impact upon scale-up is difficult to predict. The aim of this study is therefore to investigate the scalability of RED for energy generation from high concentration salinity gradients, by transitioning across three process scales: a standard 10 × 10 cm laboratory-scale stack, a 10 × 20 cm stack and a commercially available 10 × 40 cm stack. For rectangular stacks, fixing velocity doubles residence time, when length scale is increased two-fold whereas to sustain the same residence time, crossflow velocity must be doubled when length scale is increased two-fold. The scale-up response is therefore comparable to Moreno et al. [13] who studied low concentration salinity gradients (seawater/river water) in square stack design. Specific objectives are to: (i) use a widely studied low concentration gradient (seawater/river water) to benchmark the high concentration salinity gradient across three stack sizes; (ii) challenge stack sizes at these concentration gradients to compare responses to flow rate, velocity and residence time to characterise the scalability of power density at high concentration gradients; (iii) establish how energy efficiency improvements with recycle translate across stack sizes for high concentration salinity gradients; and (iv) compare the impact of increasing membrane area via stack size or cell pair number, to inform on stack design for high concentration gradients.

## 2 Materials and methods

### 2.1 Experimental setup for reverse electrodialysis stacks

Three RED stacks with dimensions of 10 × 40 cm, 10 × 20 cm and 10 × 10 cm were used in these experiments, with fluid flowing across the longest length. These stack sizes were selected on the basis that 10 × 10 cm stacks are typically used in lab-scale studies, and 10 × 40 commercial stacks are commonly available, with 10 × 20 cm selected as an intermediary to transition between these sizes. The largest stack was a commercially available RED module (RED-800-2-25, FumaTech, Bietigheim-Bissingen, Germany) equipped with Titanium mixed metal oxide electrodes. The module had a total membrane area of 2 m^2^, with 25 pairs of alternately stacked FAS-50 anion exchange and FKS-50 cation exchange membranes (Table 2), separated by 0.155 mm integrated polyester spacers. Membranes and spacers from this stack were laser cut to size and alternately stacked between custom-made acetal endplates (Model Products LT, Bedfordshire, UK) for the two smaller stack sizes. The use of the same spacers throughout the study was employed to minimise the impact of the spacer shadow effect. Titanium mixed metal oxide electrodes (MAGNETO special anodes, Schiedam, The Netherlands) were fixed inside the endplates. Peristaltic pumps delivered feed and electrode rinse solutions to the stack (Watson Marlow, Cornwall, UK). Inline conductivity meters (CDH-SD1, Omega Engineering Limited, Manchester, UK; Seven2Go Pro S7, Mettler Toledo, Leicester, UK) were fitted on the stack inlets and outlets. Feed reservoirs were placed on balances (Kern SFB 20K2HIP, Scales and Balances, Thetford, UK) to determine water flux for each cell. Stack size was initially varied at a constant cell pair number of 25 pairs, corresponding to a total membrane area of 0.5 m^2^ in the smallest stack to 2 m^2^ in the largest stack. To decouple the effect of stack size from total membrane area, stack size was also varied at a constant membrane area of 0.8 m^2^. Cell pair number was varied from 5 to 25 cell pairs in the 10 × 40 cm module.

**Table 2:** Properties of the ion exchange membrane utilised according to manufacturer specifications.

Ion Exchange Membrane	IEC (mequiv./g dry)	Permselectivity (%)	Resistance (Ω cm2)	Thickness (μm)
Fumasep FAS-50	1.6–2.0	92–96	0.6–1.5	45–55
Fumasep FKS-50	1.2–1.4	97–99	1.8–2.5	45–55

### 2.2 Preparation of solutions

Aqueous sodium chloride solutions were prepared using 99% NaCl (Alfa-Aesar, Lancashire, UK) and deionised water. A 0.51 M concentrated feed and 0.02 M dilute feed, corresponding to standard sea/river water equivalent concentrations, were prepared, in order to benchmark data form the high concentration salinity gradient. A 4 M concentrated feed and 0.02 M dilute feed were used to develop the high concentration gradient, which has been previously identified as the most suitable concentration gradient to promote high power density [23]. The electrode rinse solution contained 0.1 M K_3_Fe(CN)_6_, 0.1 M K_4_Fe(CN)_6_ (Fisher Scientific, Leicestershire, UK) and 2 M NaCl (Alfa-Aesar, Lancashire, UK).

### 2.3 Electrochemical measurements

A potentiostat (IviumStat.h, Alvatek, UK) was used to carry out electrochemical measurements, with data logged using proprietary software (IviumSoft). Feeds were pumped through the stack until a stable open circuit voltage of <0.01 V s^−1^ was obtained before beginning experiments to ensure steady-state was achieved. All experiments were carried out in triplicate. Results are reported as mean and standard deviation. For single pass experiments, chronopotentiometry, in which several current steps are utilised to derive a power density curve [9], enabled determination of optimal and maximal current and power densities. A constant current was applied at each step until the voltage stabilised. The theoretical open circuit voltage which could be achieved using ideal membranes with a 100% permselectivity was calculated using the Nernst equation [24]:

(1) $$OCV=\frac{2nRT}{zF}\ln\frac{\gamma_{C}C_{C}}{\gamma_{D}C_{D}}$$

Where n is the number of cell pairs, R is the universal gas constant (J K^−1^ mol^−1^), T is the temperature (K), z is the valency of the ion, F is the Faraday constant (C mol^−1^), γ is the mean activity coefficient of the counter-ion and C is the concentration of the counter-ion, with the subscripts C and D referring to the concentrated and dilute feeds respectively. Maximum current was determined from the roots of the power/current curve produced. Power density (P_d_, W m^−2^) was obtained from the current applied and voltage produced, and was normalised to the total active membrane area in the module:

(2) $$P_{d}=\frac{UI}{\;A}$$

Where U is the voltage (V), I is the current (A), and A is the total membrane area (m^2^). Net power was calculated to account for the power required for pumping [9]:

(3) $$P_{net}=P_{RED}-P_{p}$$

Where P_net_ is the net power (W), P_RED_ is the power produced by RED and P_p_ is the power required for pumping:

(4) $$P_{p}=\Delta p_{C}Q_{C}+\;\Delta p_{D}Q_{D}$$

Where Δp is the pressure drop (Pa) and Q is the flow rate (m³ s^−1^). The pressure drop across the channel was estimated from Ref. [25]:

(5) $$\Delta p=\frac{12\mu Lv}{h^{2}}$$

Where μ is the viscosity (Pa s), L is the channel length (m), v is the fluid velocity (m s^−1^) and h is the intermembrane thickness (m).

For experiments carried out with feeds continuously recycled, a constant current was applied until the voltage reached 0 V. Current density was normalised to the area of one electrode, with each stack tested at a range of current densities.

Energy efficiency can be calculated from the work produced by RED, W_RED_, and the total available Gibbs free energy of mixing in the system, ΔG_mix_:

(6) $$\;\eta_{RED}=\frac{W_{RED}}{\Delta G_{mix}}\;\times 100\%$$

The total work recovered can be calculated as follows:

(7) $$W_{RED}=\;\overset{t_{end}}{\underset{t_{o}}{\sum }}UI△t$$

where Δt is the time interval (s), t_0_ is the time at which current was applied and t_end_ is the time at which the voltage reached 0, and no further work was produced by the system [20]. The Gibbs equation is used to calculate the total energy available in the system:

(8) $$\Delta G_{mix}=\;\Delta G_{m}-\left(\Delta G_{c}+\Delta G_{d}\right)$$

where ΔG is the energy available in each stream (J) with the subscripts m, c and d referring to the mixed outlet stream, the concentrated and the dilute feeds, respectively. For ideal solutions, and assuming total mixing of the concentrated and dilute streams, ΔG_mix,_ is calculated from:

(9) $$\Delta G_{mix}=\;-\;\left(N_{c}+N_{d}\right)T\Delta S_{m}-\left(-N_{c}T\Delta S_{c}-\;N_{d}T\Delta S_{d}\;\right)$$

where N is the number of moles (mol), T is the temperature (K) and ΔS is the molar entropy (J K^−1^ mol^−1^). ΔS can be obtained as follows:

(10) $$\Delta S=\;-R\;\underset{i}{\sum }x_{i}ln\;x_{i}$$

where x is the mole fraction of species i.

The thermodynamic efficiency can be calculated from:

(11) $$\eta_{thermodynamic}=\frac{P_{gross}}{\left(exergy_{in}-exergy_{out}\right)}$$

## 3 Results and discussion

### 3.1 Highest gross power achieved using the largest stack size at the highest flow rates

**Fig. 1:**
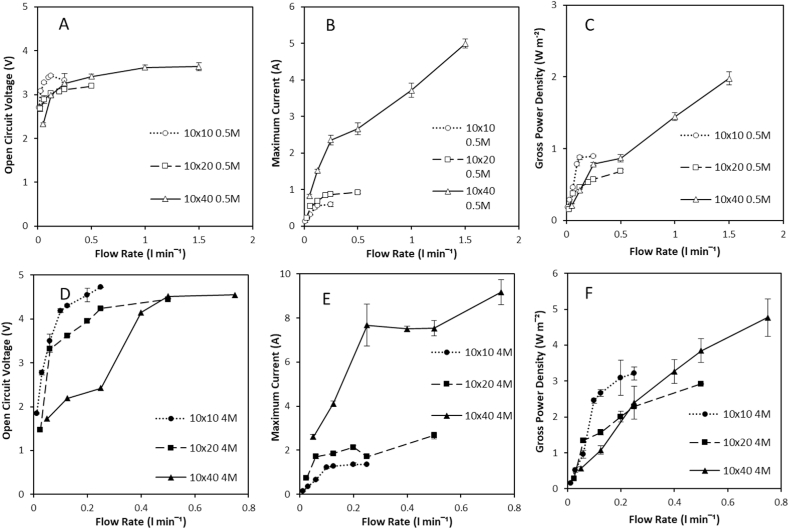
Effect of stack size and flow rate at constant cell pair number on (A) power density and (B) open circuit voltage (C) maximum current using 0.51 M and 0.02 M feeds in single pass and (D) maximum power density (E) open circuit voltage and (F) maximum current using 4 M and 0.02 M feeds in single pass. Cell pair number was fixed at 25 pairs; feed temperature, 25 °C. Error bars represent the standard deviation of a triplicate.

Three stacks (10 × 10 cm, 10 × 20 cm and 10 × 40 cm) were initially compared in single pass to evidence the effect of stack size on power density. To set a benchmark, initial experiments were carried out using a concentration gradient equivalent to seawater/river water and repeated using a 4 M feed to establish the effect of a higher concentration gradient. For seawater/river water, a minimum open circuit voltage (OCV; indicating electrochemical potential) was recorded at the lowest flow rate. Once flow rate was increased above 0.3 L min^−1^, a plateau was achieved at 3.42 ± 0.09 V for all three stack sizes (Fig. 1A), which was attributed to a reduction in concentration polarisation [26]. Notably, this plateau was reached at lower flow rates for the smaller stacks, as a result of the increasing boundary layer thickness which developed axially along the extended path length for larger stacks [22]. To illustrate, an OCV of 3.2 V was obtained using the smallest stack (10 × 10 cm, square) at a flow rate of 0.06 L min^−1^, in comparison to 2.9 and 2.3 V by the 10 × 20 cm and 10 × 40 cm rectangular stacks, respectively, at an equal flow rate. For the 4 M concentrated feed, a similar trend was observed where a higher flow rate was required for larger stack sizes to achieve a plateau for OCV of around 4.73 V (Fig. 1D). The maximum theoretical OCV estimated from the Nernst Potential (which is independent of stack size at a fixed cell pair number, Equation (1)) was 3.84 V for the 0.5 M concentrated feed, which was close to that obtained experimentally, whilst the maximum OCV obtained with the 4 M concentrated feed was significantly lower than the theoretical OCV of 6.66 V. This is likely due to the increased ionic transport [14] that resulted from the higher concentration gradient. This increases boundary layer resistance in the low concentration compartment and is exacerbated by counter-current osmotic water transport into the high concentration compartment. The combination of which lowers the salinity concentration difference across the membrane stack.

For both concentrations gradients, the current recorded at a fixed flow rate increased with stack size. This is because the increased membrane surface area facilitates greater total ionic transfer (Fig. 1B and E). For both feed concentrations, the maximum current was achieved through increasing flow rate, which can be ascribed to a reduction in concentration boundary layer effects at the membrane-fluid boundary with higher flow rates [26]. The minimisation of concentration polarisation through increased fluid velocity corresponded to a plateau in the current for the two smaller stack sizes at sea water/river water concentrations, as maximum ionic transport was achieved. However, for the 10 × 40 and the stacks utilising 4 M/0.02 M, increasing the flow rate continued to produce an increase in current, due to the increased ionic transport across the membranes facilitated by the higher concentration gradient, increase in concentration gradient and for the 10 × 40, increased membrane area. The maximum current accords with the gross power density (P_d_) recorded for the largest stack which was equivalent to 1.98 W m^−2^ and 4.77 W m^−2^ for the 0.5 and 4 M feed concentrations respectively. However, below a feed flowrate of 0.25 L min^−1^, the highest power density was obtained in the smallest stack (Fig. 1C). For example, at a feed rate of 0.25 L min^−1^, power densities of 3.2, 2.4 and 2.3 W m⁻^2^ were obtained for the 10 × 10 cm (square), 10 × 20 cm and 10 × 40 cm (rectangular) stacks respectively using the 4 M feed concentration (Fig. 1F). This discontinuity was explained by the cumulative effect of the increased current in larger stacks, which then introduced a greater boundary layer resistance in the low concentration compartment, subsequently reducing stack voltage. Since the length scale increases in only one dimension for rectangular stacks, a fixed flow rate for each stack size is equivalent to fixing velocity. When the path length is short, high power densities are obtained despite the lower current, as concentration polarisation is negligible which increases the voltage. Conversely, once the path length is extended at an equivalent velocity, the current increases due to the increase in membrane surface area and longer solution residence time. However, this drives the development of a more concentrated boundary layer when path length is extended, subsequently diminishing the potential such that lower gross power densities are achieved for these conditions at larger stack sizes. Whilst this effect is exacerbated for high concentration gradients, higher gross power densities are eventually observed for larger stacks following an increase in flow rate above around 0.4 L min^−1^ (Fig. 1F).

**Fig. 2:**
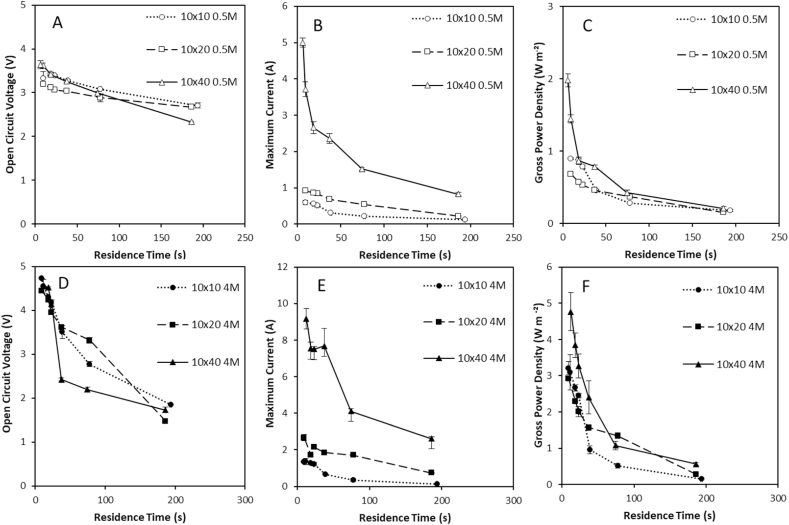
Effect of stack size and residence time at constant cell pair number on (A) power density and (B) open circuit voltage (C) maximum current using 0.51 M and 0.02 M feeds in single pass and (D) maximum power density (E) open circuit voltage and (F) maximum current using 4 M and 0.02 M feeds in single pass. Cell pair number was fixed at 25 pairs; feed temperature, 25 °C. Error bars represent the standard deviation of a triplicate.

To compensate for the longer path length that is introduced with larger stacks, Moreno et al. [14] proposed that residence time could be used to achieve comparable performance between square RED stacks for seawater/river water concentration gradients. When doubling the length of the stack in this study from 10 × 10 cm (square) to 10 × 20 cm (rectangular), flow rate must be doubled (2Q) to fix an equivalent residence time, which consequently doubles feed velocity. For power densities to match across stack sizes, two assumptions are therefore fostered: (i) at an equivalent residence time, ionic flux scales to membrane area across stack sizes [14]; and (ii) the increased polarisation phenomenon introduced by the longer stack length may be compensated for by the higher feed side velocity imposed. For the low concentration gradient, similar OCVs were obtained for each stack size at a constant residence time when a 0.5 M concentrated feed was used (Fig. 2A). For residence times in the range 30–190s, the maximum current increased approximately in proportion to the stack membrane area (Fig. 2B). Consequently, power densities recorded for the low concentration gradient within this range of residence times were comparable for each stack size (Fig. 2C), which corroborates previous work on scaling-up RED for seawater/river water [14]. For residence times below 30s, divergence of the power density data was evident, due to the disproportionate increase in current created by the largest stack size. This can be accounted for by the significantly higher feed velocity applied at comparable residence times, which reduced concentration polarisation to improve ionic transport. For the high concentration salinity gradient, OCV was similar across stack sizes for residence times <20s (Fig. 2D). However, for longer residence times, a considerably lower OCV was recorded for the large stack. Whilst current was higher for the 4 M feed, the trend was comparable to the low concentration gradient where an approximately proportionate relationship between membrane area and current was identified between residence times of 80 and 190s. However, power densities were not comparable between stack sizes, indicating that high ionic transport and water transport [23] introduce complex polarisation phenomena which make scale-up of RED for energy generation from high concentration gradients difficult to predict (Fig. 2F).

### 3.2 Net power density and energy efficiency trade-off when scaling-up in single pass

**Fig. 3:**
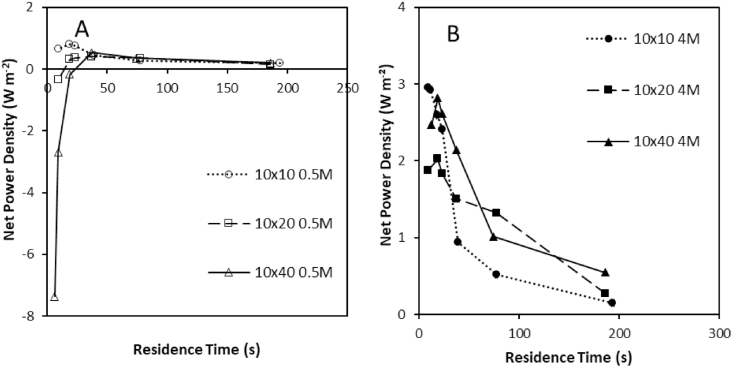
Effect of stack size and flow rate on net power density for (A) 0.5 M concentrated feed and (B) 4 M concentrated feed in single pass. Cell pair number was fixed at 25 pairs; feed temperature, 25 °C.

The highest gross power density was recorded at the shortest residence time for the 10 × 40cm stack (Fig. 2C). However, due to the increased pressure drop imposed by higher flow rates (4Q versus the 10 × 10cm) and longer channel length within the larger stack, a negative net power density was recorded for the 0.5 M feed concentration when residence time was <20s, making it impractical for implementation (Fig. 3A). In contrast, for the 4 M feed concentration, net power density was consistently positive for all residence times; the highest net power density was recorded at the shortest residence time (Fig. 3B). This is because the concentration gradient promoted a high electrochemical potential, whilst the relative increase in fluid velocity that corresponds to a decrease in residence time, reduced boundary layer thickness subsequently improving ionic transport [20,27]. Whilst gross power density was considerably higher for the 10 × 40cm stack with the 4 M feed concentration, comparison between the 10 × 10cm and 10 × 40cm stacks demonstrates comparable net power densities for the 4 M feed concentration due to the increased pressure drop of the larger stack (Fig. 2F) [20,27]. Consequently, the comparative normalised membrane cost (€ kWh^−1^) between the small and large stacks can be considered comparable; the choice of stack size is therefore likely to be based on a systems engineering approach related to the economies of scale for stack manufacture, the relative capital cost for pumping and the reduced total current provided by the small stack.

**Fig. 4:**
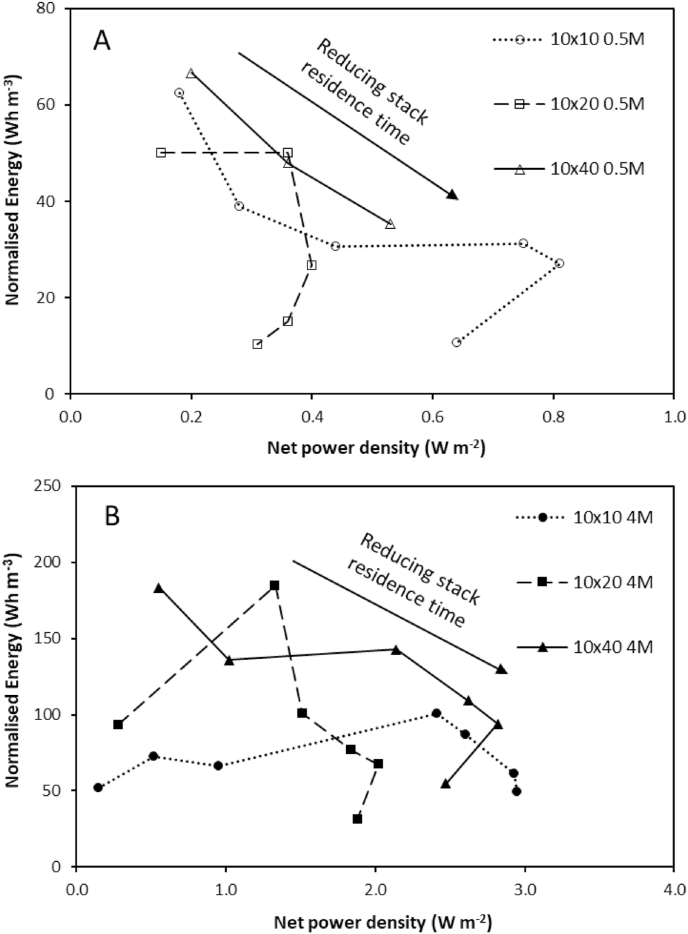
Normalised energy and net power density for (A) 0.5 M concentrated feed and (B) 4 M concentrated feed in single pass at varying residence times. Cell pair number was fixed at 25 pairs; feed temperature, 25 °C.

For closed-loop applications, the high net power density observed at short residence times, implies a trade-off between system efficiency and the capital cost required for power generation, to minimise exergy destruction and improve efficiency of the combined system (RED with regenerative step). This is illustrated by comparing the net energy generated per unit of feed (Wh m^−3^) versus the net power density created at each residence time studied (Fig. 4). Peak power density is realised at the shortest residence time that corresponds to the lowest volumetric energy recovery, and conversely the minimum power density is recorded for the residence time which provides the highest volumetric energy recovery. Whilst not definitive, the largest stack size potentially provides the greatest trade-off between power and energy. However, an alternative perspective is to approach stack design and stack configuration for closed loop application as a classical mass transfer problem [28]:

(12) $$\frac{C}{C_{0}}=e^{-\frac{kal}{v}}$$

which identifies that in order to maximise utilisation of the feedside concentration ($C/C_{0}$), the mass transfer coefficient (k, m s^−1^) is first optimised through limiting the boundary layer thickness, by increasing fluid velocity (short residence time) to improve power density, followed by extending path length in order to increase residence time (l/v, s), which will improve energy recovery. To maximise net power density from a single stack, and extend path length, several approaches can be considered: (i) increasing the number of RED stacks in series, or (ii) recycling the feed to reduce unused exergy leaving the stack [20,29]. A similar design approach was proposed by Weiner et al. [30] for RED systems using sea and river water feeds to minimise the unitary cost of pre-treatment. Whilst pre-treatment is not required for closed-loop application, this work indicates that such a strategy is also applicable to high concentration salinity gradients brines to maximise systems level energy efficiency.

### 3.3 Exergy dissipation can be controlled by stack size and current density in recycle

**Fig. 5:**
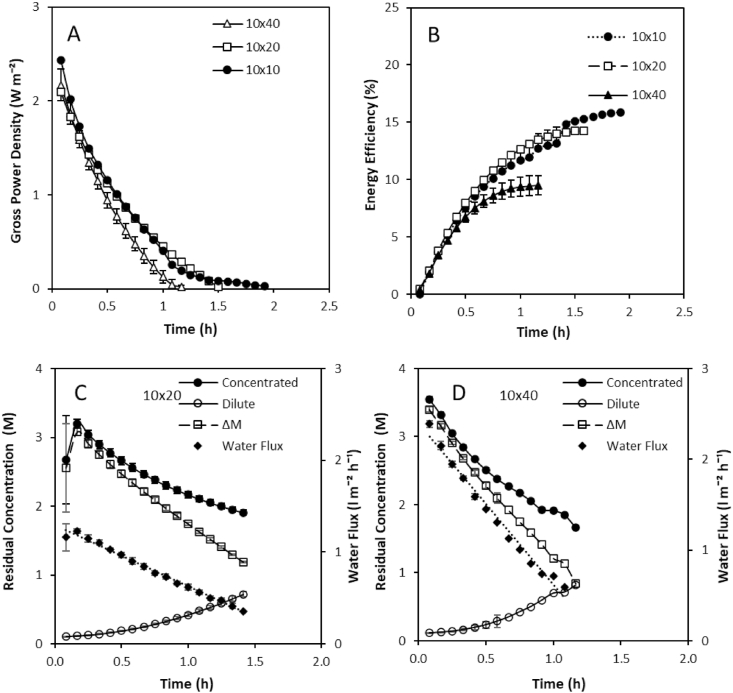
Effect of stack size on (A) Gross power density over time and (B) energy efficiency over time from 4 M to 0.02 M feeds in recycle. Feed volume was normalised to membrane area; residence time fixed at 20s; current density, 40 A m⁻^2^; feed temperature, 25 °C. Concentration profile and water flux at stack size of: (C) 10 cm × 20 cm; and (D) 10 cm × 40 cm.

A high concentration gradient (4 M and 0.02 M) was established across the three stacks, and the solutions operated in recycle to maximise thermodynamic efficiency (Equation (12)). The electrochemical potential declined as feeds were recirculated (Fig. 5A), comparable to the discharge of a battery [29]. Energy efficiency subsequently improved during discharge, which reflected the increased utilisation of the concentration gradient (Fig. 5B). The discharge curve was characterised by two phases, an initial linear decline in energy generation, followed by a non-linear phase, which terminated in a plateau (Fig. 5B). At a fixed current density (40 A m⁻^2^) and residence time (20s), the smallest stack achieved the highest energy efficiency. The higher exergy losses for larger stack sizes was due to the increased osmotic water transport, induced by the increase in membrane surface area (Fig. 5C and D). For both stacks, power production terminated whilst a high residual feed concentration remained (around 2 M). This was attributed to the effect of concentration polarisation in the boundary layer of the dilute feed compartment, which was induced by the osmotic transport of water from the dilute feed to the concentrated feed, subsequently negating the concentration driving force and leading to a termination in energy generation.

**Fig. 6:**
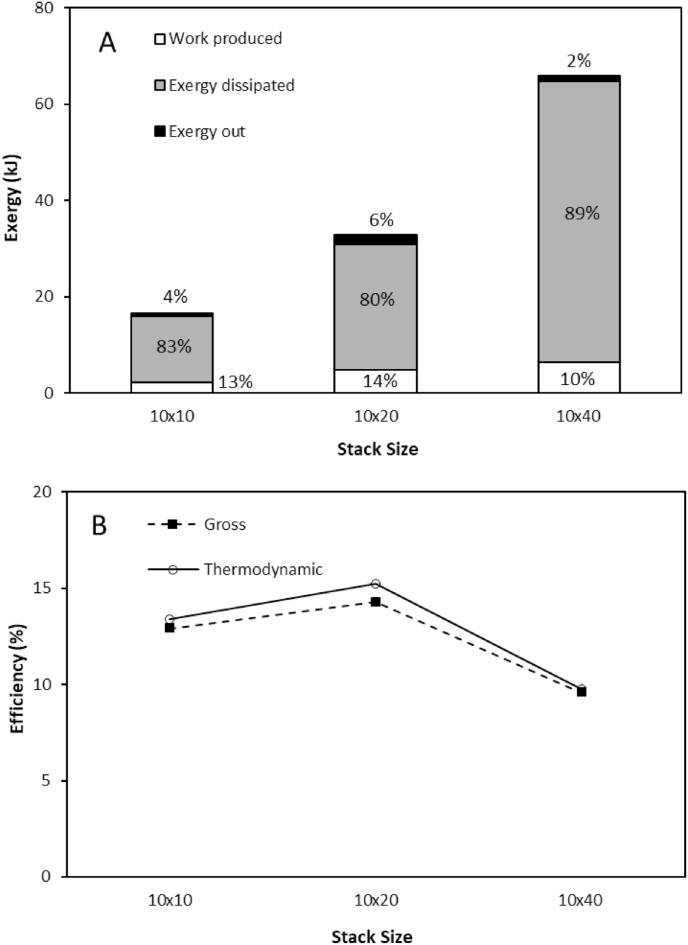
(A) Exergy analysis and (B) gross and thermodynamic efficiency obtained by the three stack sizes from 4 M to 0.02 M feeds in recycle. Feed volume was normalised to membrane area; residence time fixed at 20s; current density, 40 A m⁻^2^; and feed temperature, 25 °C. Error bars represent the standard deviation of a triplicate.

**Fig. 7:**
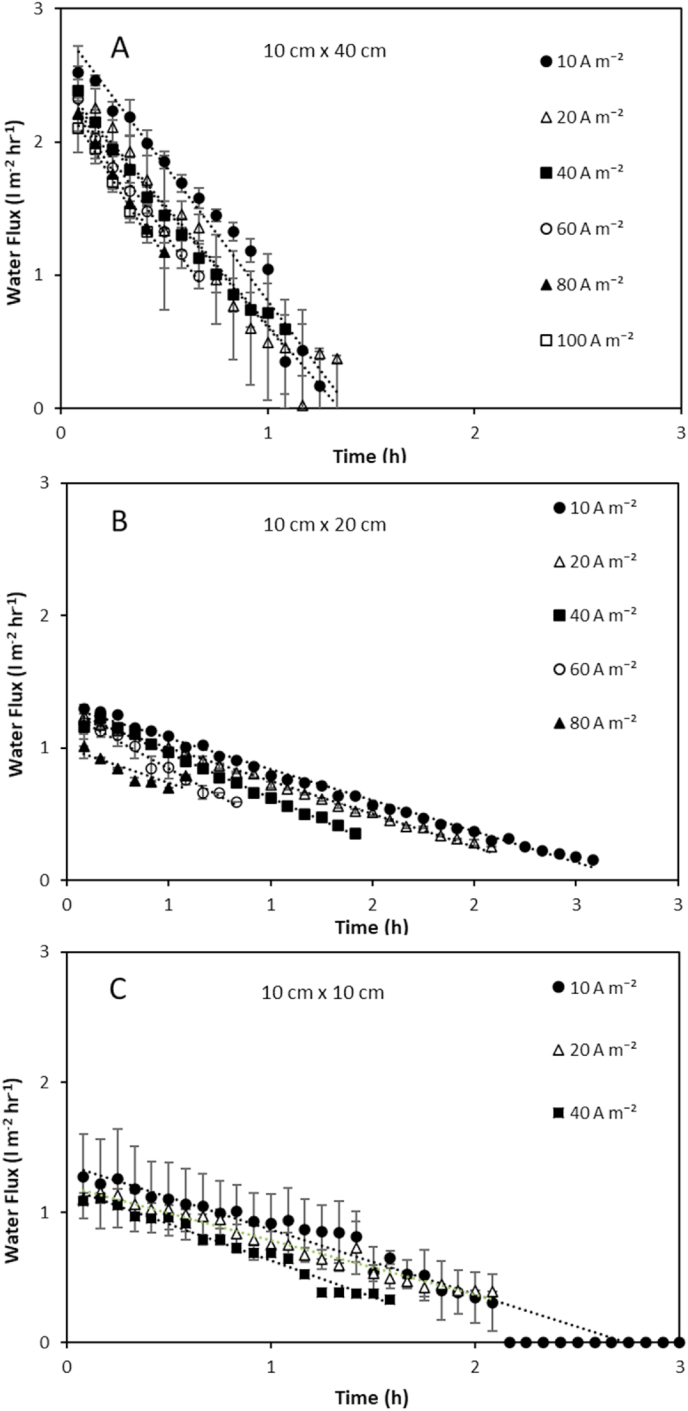
Effect of current density on water flux over time for (A) 10 cm × 40 cm (B) 10 cm × 20 cm and (C) 10 cm × 10 cm at fixed cell pair number. Feed volume was normalised to membrane area and residence time fixed at 20s. Cell pair number was fixed at 25 pairs; feed temperature, 25 °C. Error bars represent the standard deviation of a triplicate.

**Fig. 8:**
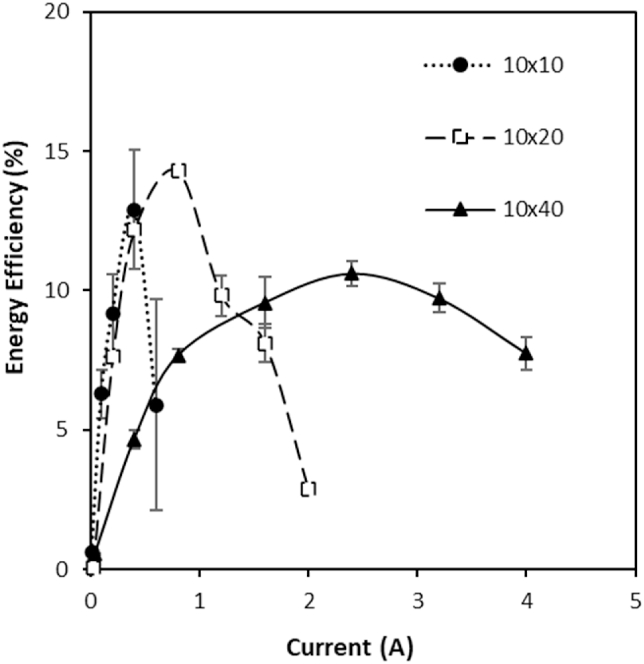
Effect of stack size and current on energy efficiency from 4 M to 0.02 M feeds in recycle. Feed volume was normalised to membrane area and residence time fixed at 20s. Cell pair number was fixed at 25 pairs; feed temperature, 25 °C; current density, 40 A m⁻^2^. Error bars represent the standard deviation of a triplicate.

An exergy analysis of the three stacks demonstrated that recycling feeds minimised the unused exergy in the effluent. As recycling the feeds minimised the unused exergy leaving the stack, thermodynamic efficiency was similar to the gross energy efficiency in this study (Equation (6)). However, the majority of the exergy provided to each stack was dissipated and not used for power production (Fig. 6a). These exergy losses were greatest for the largest stack at 89%, resulting in just 10% of the available energy being utilised for power production, compared to 13 and 14% in the 10 × 10 and 10 × 20 stacks, respectively. Moreno et al. [14] similarly determined an increase in exergy loss due to water flux and co-ion transport for larger stacks operated with seawater and river water feeds in single pass at an equal velocity, with 55% of exergy dissipated in the 44 cm × 44 cm stack compared to 15% in a 6 × 6 cm stack. Increased exergy losses can be anticipated in this study since the concentration gradient is increased, producing greater water and co-ion transport [21,29]. However, in this study, the increase in water flux (normalised for membrane surface area) was not proportional to membrane surface area, and instead increased for larger stack sizes (Fig. 7). This can be explained by the higher velocity required for the largest stack size to sustain a comparable residence time, which was four times greater for the 10 × 40cm compared to the 10 × 10cm stack. Water transport from the dilute feed compartment by osmosis, dilutes the salt concentration within the high concentration compartment boundary layer. The high salinity concentration gradient is then re-established through surface renewal within the boundary layer, which serves to enhance osmotic water transport at the higher velocities. This effect is exacerbated for high concentration gradients with larger surface area stacks due to the higher ionic transport imposed (Fig. 5c,d). The highest energy efficiency was therefore identified for the smaller stacks (Fig. 6b), due to the lower exergy losses created by water transport. This contradicts the work of Moreno et al. [14] in which gross efficiency was observed to increase with stack size for seawater river water mixing, the delineation between studies being the concentration gradient applied. However, increasing current density decreased water flux for all stack sizes (Fig. 7), suggesting that an increase in electro-osmosis counteracts osmotic water transport [29] and is therefore critical to the development of RED stacks for high concentration salinity gradients. The optimum current required to achieve peak energy efficiency increased with stack size (Fig. 8), which is likely due to the greater ionic transport facilitated by larger stacks at an equivalent residence time [14]. Consequently, given the relatively small difference in thermodynamic efficiency between stack sizes, comparative efficiency maybe realised for larger RED stacks through optimising current density for high concentration gradient RED, to take advantage of the reduction in boundary layer resistance accommodated by the increase in current density [31].

### 3.4 Power density increases as cell pair number is increased for concentrated brines

**Fig. 9:**
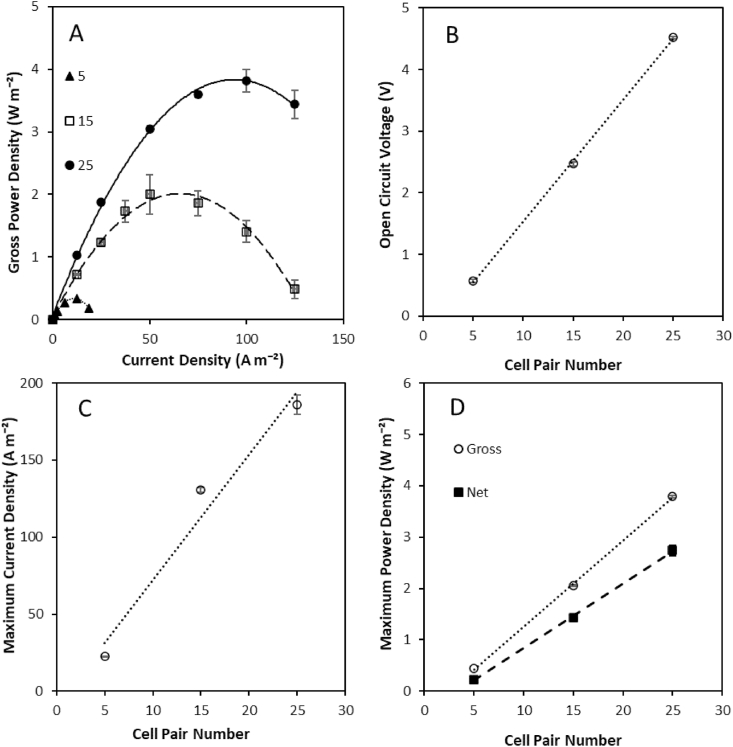
Effect of varying cell pair number on (A) power density and current density; (B) open circuit voltage; and (C) maximum current density and (D) maximum gross and net power density obtained using 4 M and 0.02 M feeds in single pass in a 10 cm × 40 cm RED stack. Residence time fixed at 20s; and feed temperature, 25 °C. Error bars represent the standard deviation of a triplicate.

Cell pair number was varied in the largest stack (5–25 cell pairs) to establish the impact on power density and energy generation from a high concentration gradient. Open circuit voltage increased linearly with an increase in cell pair number, in accordance with the Nernst Equation (Fig. 9). Current density (normalised to the area of one electrode) across the stack also increased with cell pair number (Fig. 9), which is to be expected since electrical current is proportional to the voltage and inversely proportional to the resistance. The proportional increase in power with increasing cell pair number from 5 to 25 also suggests the electrode system was not limiting to power production at this number of repeating pairs, an issue which could occur during system scale up, beyond such stack sizes. The increased current in this study is therefore associated with the increase in flow rate applied to compensate for the additional cell pairs in order to sustain a comparable residence time across the stack. By scaling cell pair number on residence time, an equivalent velocity is sustained and therefore a similar impact on flow conditions on the boundary layer (non-ohmic) resistance can be assumed. However, net power density increased with cell pair number (Fig. 9). This was attributed to a non-proportional increase in optimum current density with cell pair number and is comparable to previous observations where current density was optimised for cell pair number [32]. In most examples of RED utilising natural salinity gradients, the total area resistance is assumed to be independent of current density. However, it has been shown that this is not true with the largest reduction in resistance occurring in the diffusion boundary layer at high current densities [31]. We propose that the higher current density applied across the stack at larger cell pair numbers, advantaged power generation by reducing osmotic water transport due to the increased electro-osmosis which occurs at higher current densities, thereby limiting boundary layer effects, which has been previously demonstrated for fixed stack sizes [29]. This is, however, much more significant when using a high concentration gradient (Fig. 7) due to the exponential increase in osmotic pressure leading to greater osmotic water transport and ion transport. The practical significance is that through increasing cell pair number at optimum current density, there may be opportunity to simultaneously improve both power density and energy efficiency of a single RED stack for high concentration salinity gradients, through minimising boundary layer resistance.

## 4 Conclusions

In this study, the scalability of RED stack design was investigated for energy generation from high concentration salinity gradients. The following conclusions were drawn:

• An increase in fluid velocity was required to optimise gross power density as stack size increased, due to the more concentrated boundary layer that developed axially along the length of the larger stack. At an equivalent stack size, a greater velocity was required to improve OCV for the high concentration salinity gradient; both concentration gradients achieving a plateau as flow rate was increased.
• Whilst the OCV obtained for the low concentration gradient was comparable to the theoretical OCV, the theoretical OCV was not achieved for the high concentration gradient. This manifests from the greater boundary layer resistance experienced in the dilute compartment. This is attributed to the higher ionic flux from high to low concentration gradient, coupled with counter-current water transport into the high concentration compartment, the combination of which reduces the local concentration gradient across the membrane to drive mass transport.
• Negative net power densities were recorded for larger RED stacks using seawater/riverwater saline conditions due to the increased pumping power demand, indicating small stacks benefit energy generation from low concentration salinity gradients. For higher concentration gradients, the pressure drop is compensated for by the increased ionic flux, and so short residence times are favoured for maximising net power density, independent of stack size.
• Whilst comparable power densities are obtained from small and large RED stacks, the underlying mechanism is distinct. Short stacks sustain high OCV by minimising water transport, whilst longer stacks facilitate higher current due to the increased surface area. Consequently, there may be a trade-off in determining the best module size for scale-up that may be more focussed on a systems approach to cost minimisation (e.g. minimising pump cost), that provides the ultimate decision. However, larger stacks may be preferred due to the high current, complemented by an improved thermodynamic efficiency as demonstrated in single pass.
• Increasing cell pair number improved power density and single stack energy efficiency due to the increase in maximum current density, which reduces boundary layer resistance; an effect particularly observed for high concentration salinity gradients. Consequently, improvements to thermodynamic efficiency and power density may be obtained for single RED stacks applied to high concentration salinity gradients through optimising for cell pair number.
• Greater volumetric exergy conversion was attained in recycle, which is critical for closed-loop RED applications. Whilst smaller stacks demonstrated lower exergy dissipation, optimisation of current density for larger stacks to reduce water transport, could be sufficient to achieve comparable or better thermodynamic efficiencies.

## Author statement

A.M. Hulme: Conceptualization; Data curation; Formal analysis; Investigation; Methodology; Validation; Roles/Writing - original draft.

C.J. Davey:Conceptualization; Data curation; Formal analysis; Investigation; Methodology; Resources; Validation; Writing - review & editing.

S. Tyrrel: Funding acquisition; Project administration; Resources; Writing - review & editing.

M. Pidou: Conceptualization; Data curation; Formal analysis; Resources; Writing - review & editing.

E.J. McAdam: Conceptualization; Data curation; Formal analysis; Funding acquisition; Investigation; Project administration; Resources; Supervision; Writing - review & editing.

## Declaration of competing interest

The authors declare that they have no known competing financial interests or personal relationships that could have appeared to influence the work reported in this paper.
